# Interaction of *Panax quinquefolius* Saponin and Dual Antiplatelets on Vascular Endothelial Function in Rats with Acute Myocardial Infarction

**DOI:** 10.1155/2015/932751

**Published:** 2015-05-19

**Authors:** Baojun Wang, Yue Liu, Qinghua Shang, Qingxiang Zhang, Lei Zhang, Jiangang Liu, Dazhuo Shi

**Affiliations:** ^1^Oriental Hospital Affiliated to Beijing University of Chinese Medicine, Beijing 100091, China; ^2^Centre of Cardiovascular Diseases, Xiyuan Hospital of China Academy of Chinese Medical Sciences, Beijing 100091, China; ^3^China Heart Institute of Chinese Medicine, China Academy of Chinese Medical Sciences, Beijing 100091, China; ^4^Emergency Department, Xiyuan Hospital of China Academy of Chinese Medical Sciences, Beijing 100091, China

## Abstract

The objective of this study is to investigate the interaction of *Panax quinquefolius* saponin (PQS) and dual antiplatelets (aspirin and clopidogrel) on antiplatelet activity and vascular endothelial function in rats with acute myocardial infarction (AMI). Forty-eight male SD rats were randomly designed into sham group, model group, dual antiplatelet group, and PQS plus dual antiplatelet group. AMI rats were induced by ligation of left anterior descending coronary artery (LAD) and dual antiplatelet agents and additional PQS to dual antiplatelets were intragastrically administered for 28 days, respectively. The ventricular cavity area and cardiac transverse area ratio in PQS + dual antiplatelet group showed a decreased tendency. PAgT(%) decreased significantly in both dual antiplatelet group and PQS + dual antiplatelet group. TXB_2_ concentration significantly decreased in dual antiplatelet and PQS + dual antiplatelet groups, whereas 6-keto-PGF1_*α*_ concentration significantly increased in PQS + dual antiplatelet group. Rats in PQS + dual antiplatelet group demonstrated a significant decrease in plasma ET-1 concentration and an increase in serum NO concentration compared with dual antiplatelet group. The combination therapy of PQS and dual antiplatelets showed some beneficial effects on vascular endothelial function and ventricular remodeling in rats with AMI.

## 1. Introduction

Cardiovascular diseases remain the predominant cause of morbidity and mortality all over the world. Immediately percutaneous coronary intervention (PCI) is one of the most frequently therapies used in clinical practice for acute myocardial infarction (AMI), which remarkably increased the successful rate of revascularization and reduced the mortality and morbidity of AMI patients [1, 2]. Despite the fast development of interventional cardiology, drug therapy has always been a cornerstone in the treatment of cardiovascular diseases. Based on the integrative medicine of Eastern and Western worlds, the application of herbal medicine (HM) has valuable significance in reducing the risk of cardiovascular event [3]. In Asia, HM is used for prevention and treatment of coronary heart disease (CHD) in many countries for thousands of years. Many studies showed that the combination therapy of HM and conventional western medicine significantly improved life quality and prognosis of patients after PCI [4, 5]. Clinical application of dual antiplatelet drugs such as aspirin and clopidogrel is important in the prevention and therapy of CHD, but prolonged treatment with dual or triple antiplatelet drugs revealed a diversity of platelet reactions, which limits the clinical application widely.

*Panax quinquefolius* saponin (PQS) is an active part extracted from the stems and leaves of* Panax quinquefolius* (American ginseng), which has a beneficial effect on the treatment of CHD with increasing energy storage in ischemic myocardium, promoting angiogenesis, inhibiting oxidative stress injury and ventricular remodeling, and so forth, just as indicated in our previous studies [6–8]. However, whether PQS has some additional beneficial effects with dual antiplatelets (aspirin and clopidogrel) in thrombosis, antiplatelet activity and improving endothelial function for CHD patients remain unclear. This study was designed to examine the interaction of PQS and dual antiplatelets (aspirin and clopidogrel) on antiplatelet activity and vascular endothelial function in rats with AMI.

## 2. Methods

### 2.1. Animals Grouping and Treatment

Male Sprague-Dawley rats (weight 200–220 g) were purchased from Beijing University Laboratory Animal Center (the animal certificate number: SCXK (Jing) 2011-0004). Rats were housed in humidity-controlled (60 ± 10)% rooms at (24 ± 1)°C with a 12 h on/12 h off light cycle. The animals were maintained with free access to standard diet and tap water.

After one week of adaptive feeding, AMI model was created in rats by ligating the left anterior descending coronary artery (LAD) as described before [9]. Twelve rats without AMI were assigned to sham group, and rats with successful operation of coronary artery ligation were divided randomly into 3 groups as follows, model group; dual antiplatelets group (9 mg/kg/d of aspirin and 6.75 mg/kg/d of clopidogrel); PQS + dual antiplatelets group (Xinyue capsules (162 mg/kg/d) combined with dual antiplatelets), with 12 rats in each group. All drugs were diluting with distilled water, and the dosages were evaluated with body surface coefficient conversion between human and rat. Rat weights were measured every week. Rats in sham and model groups were administered with the same volume of distilled water. All rats were intragastrically administrated for 28 days. The Animal Care and Use Committee of Xiyuan Hospital of China Academy of Chinese Medical Sciences approved the experimental protocol.

### 2.2. Drugs

Xinyue capsules (50 mg PQS/capsule) were purchased from Yisheng Pharmaceutical Co., Ltd., Jilin Province of China (SFDA Approval number Z20030073); clopidogrel hydrogen sulfate tablets (75 mg/pill) were purchased from Sanofi Winthrop Industry, France (SFDA Approval number J20080090); aspirin enteric-coated tablets (50 mg/pill) were purchased from Peking Shuguang Pharmaceutical Co., LLC (SFDA Approval number H11020827); and penicillin sodium injection (80 IU/bottle) was purchased from North China Pharmaceutical Co., Ltd. (SFDA Approval number X1105313). All the drugs were dissolved before use.

### 2.3. Thrombosis Time

Six rats were chosen randomly in every group to test thrombosis time. After the last intragastric administration, rats were anesthetized with intramuscular 4% chloral hydrate (0.9 mL/100 g body weight) solution and disinfected for operation. The skin was sheared at the right side of cervical part. The length of right carotid artery exposed was about 2 cm with padded cellophane under the vessel to protect the surrounding tissues. Stimulating electrode and temperature reporter were placed under the vessel separated to stimulate vascular by current of 80 *μ*A for 7 min, resulting in thrombosis. The sudden temperature decreases at the distal vascular detected by temperature reporter meant thrombosis. The thrombosis time, also known as occlusion time, was measured from the beginning of simulation to thrombosis.

### 2.4. Thrombus

As described before [10], the length, wet weight, and dry weight of thrombus were tested in the other 6 rats* in vitro*. Blood sample was drawn out from abdominal aortic and then injected into silicone tube of 2 mm diameter and 25 cm length quickly to reach about 1/2 length of the tube. The silicone tubes were put into Thrombosis and Platelet Adhesion Instrument (XSN-R11, Wuxi 2nd Electrics and Electronics Factory, Jiangsu province) and uniformly rotated for 15 min. Thrombus was picked out of the tubes to measure the length and wet weight after absorbing liquid gently with filter paper. The dry weight of thrombus was tested after heating in dryer at 37°C for 20 min.

### 2.5. Detection of Platelet Aggregation

About 2 mL of blood sample was drawn out from abdominal aortic and mixed with 3.8% sodium citrate (v/v = 9 : 1). Platelet-rich plasma was obtained by centrifuging at 800 r/min for 10 min, while platelet-poor plasma was obtained by centrifuging at 3000 r/min for 10 min. Concentration of platelet was adjusted in PRP to 600–700 × 10^9^/L. According to Born's method [11], platelet aggregation was induced by adenosine diphosphate (ADP), and the maximum platelet aggregation rate was measured by Automatic Four-channel Platelet Aggregation Instrument (LBY-NJ2, Shanghai Precil Instrument, Inc.).

### 2.6. Concentration of Thromboxane B_2_ (TXB_2_) and 6 Ketone Prostaglandin F_1*α*_ (6-Keto-PGF_1*α*_)

The concentrations of TXB_2_ and 6-keto-PGF_1*α*_ in plasma were detected with radioimmunoassay (r-911, Industrial Corporation of China University of Science and Technology) according to the procedures of kits specification provided by Beijing Huaying Biotechnology Research Institute.

### 2.7. Concentration of Endothelin-1 (ET-1) and Nitric Oxide (NO)

ET-1 concentration in plasma was measured by radioimmunoassay according to the procedures of kits specification (British Enzo Co.). NO level in serum was detected with nitrate reductase method using automatic microplate reader (MK3, Finland Labsystems Co.) referring to as the kit instruction (British Enzo Co.).

### 2.8. Ratio of Ventricular Cavity Area and Cardiac Transverse Area

After blood sample was drown out from abdominal aortic, heart was removed quickly. Cardiac cavity was irrigated by saline and liquid on surface was absorbed. Pathological section was cut into 5 pieces (5 mm in each piece) down from the ligation and parallel with coronary ditch. Sections were stained with haematoxylin-eosin (HE), and ventricular cavity area and cardiac transverse area were measured and the ratios between them were calculated by color pathological image analysis system (DpxView Pro, Denmark DeltaPix Co.). Pathological changes of rat myocardial tissues were observed by optical microscopy (BN-2, Japan Olympus Co.).

### 2.9. Coagulation Related Markers

Semiautomatic blood coagulation instrument (produced by Teco MedicalInstrumentsProduction, Germany; supplied by Beijing Chuangxin technology Co., LTD.) was used for measuring APTT (kit number A04-080929-N05), PT (kit number V28417), TT (kit number 011-110397), and FIB (kit number S210028) following the introduction of each kit.

### 2.10. Statistical Analysis

Statistical analysis was performed using SPSS 17.0 software. The mean ± standard deviation (SD) was determined for each group. Statistical analysis was performed with ANOVA *F* test and the nonparametric Kruskal-Wallis test. Differences were considered statistically significant when *P* < 0.05, and the exact *P* values were shown unless *P* < 0.001.

## 3. Results

### 3.1. The Ratio of Ventricular Cavity Area and Cardiac Transverse Area

**Figure 1:**
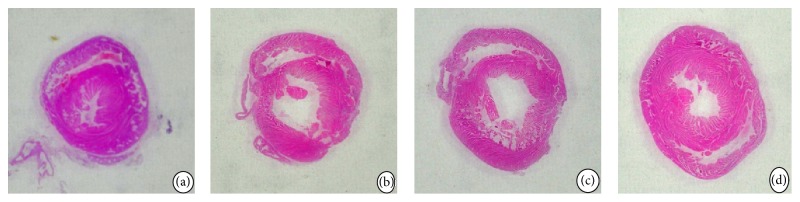
Cardiac transverse section in different groups (HE staining, general pathologic). (a) Sham group; (b) model group; (c) dual antiplatelets group; (d) PQS + dual antiplatelets group.

**Figure 2:**
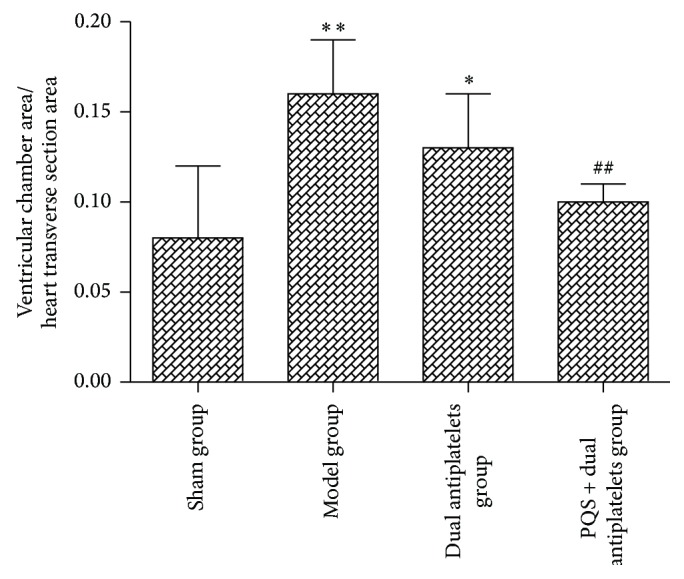
The ratio of ventricular cavity area and cardiac transverse area in different groups. The ratio was significantly lower in the PQS + dual antiplatelets group than in the model group. Data are mean ± SD (*n* = 12 rats/group). ^*^ *P* < 0.05 and ^**^ *P* < 0.01 versus sham group, ^##^ *P* < 0.01 versus model group.

Rats in model group showed thinned ventricular wall in infarction area, thickened ventricular wall in noninfarctional area, ventricular cavity dilatation, deformation and ventricular cavity area and heart transverse section ratio significantly increased compared with sham group (*P* < 0.001). Ventricular wall changes had been improved to different degree in all intervention groups, and the ratio of the ventricular cavity area and cardiac transverse area was decreased significantly in PQS + dual antiplatelets group (*P* = 0.001) compared with model group. The ventricular cavity area and cardiac transverse area ratio in PQS + dual antiplatelets group showed a decrease tendency, but there was no statistical difference (*P* = 0.058) compared with dual antiplatelets group (see Figures 1 and 2).

### 3.2. Concentrations of Plasma ET-1 and Serum NO

**Figure 3:**
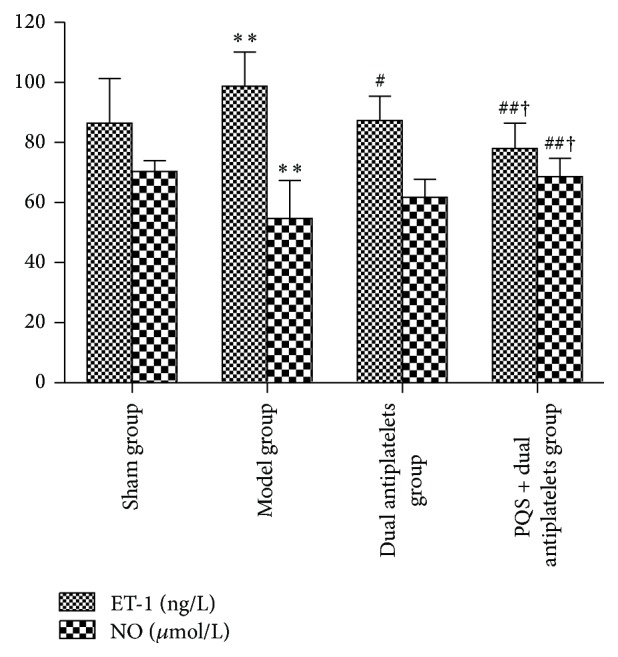
Endothelial function in different groups. Plasma ET-1 concentration was significantly decreased in the dual antiplatelets group and PQS + dual antiplatelets group compared with the model group, and it was also significantly decreased in the PQS + dual antiplatelets group compared with the antiplatelets group. Serum NO concentration was significantly increased in the PQS + dual antiplatelets group compared with the model group and dual antiplatelets group. Data are mean ± SD (*n* = 12 rats/group). ^*^ *P* < 0.05 and ^**^ *P* < 0.01 versus sham group, ^#^ *P* < 0.05 and ^##^ *P* < 0.01 versus model group, and ^†^ *P* < 0.05 versus dual antiplatelets group.

Compared with sham group, plasma ET-1 concentration increased remarkably (*P* = 0.009), whereas serum NO concentration decreased significantly in model group (*P* = 0.009). Plasma ET-1 concentration showed a significant decrease in PQS + dual antiplatelets group (*P* < 0.001), while serum NO concentration showed a significant increase in PQS + dual antiplatelets group (*P* < 0.001) and an increased tendency in dual antiplatelets group (*P* = 0.059) compared with model group. In addition, a decreased level of plasma ET-1 and an increased level of serum NO have been found in PQS + dual antiplatelets group compared with dual antiplatelets group (*P* = 0.047, *P* = 0.047) (see Figure 3).

### 3.3. Platelet Aggregation Rate (PAgT(%))

**Figure 4:**
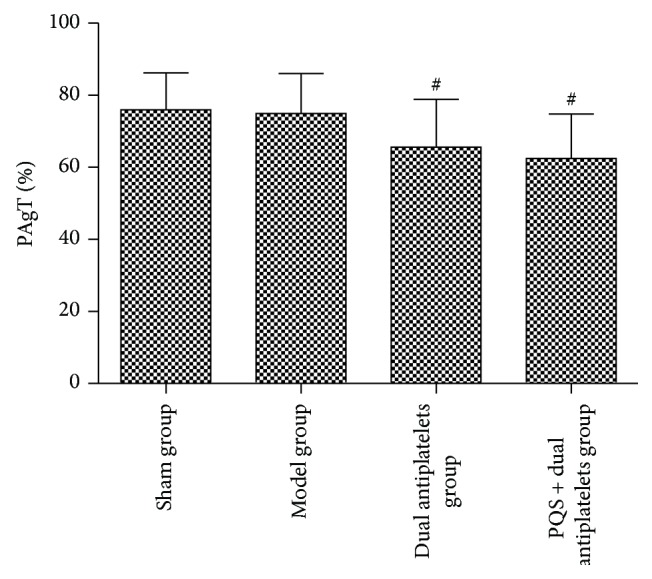
PAgT(%) in different groups. PAgT(%) decreased significantly in both dual antiplatelets group and PQS + dual antiplatelets group compared with the model group; data are mean ± SD (*n* = 12 rats/group), ^#^ *P* < 0.05 versus model group.

PAgT(%) decreased significantly in both dual antiplatelets group and PQS + dual antiplatelets group (*P* = 0.039, *P* = 0.013) compared with model group. PQS + dual antiplatelets group showed no statistical difference in PAgT(%) (*P* = 0.515 > 0.05) compared with dual antiplatelets group (see Figure 4).

### 3.4. Thrombosis

**Figure 5:**
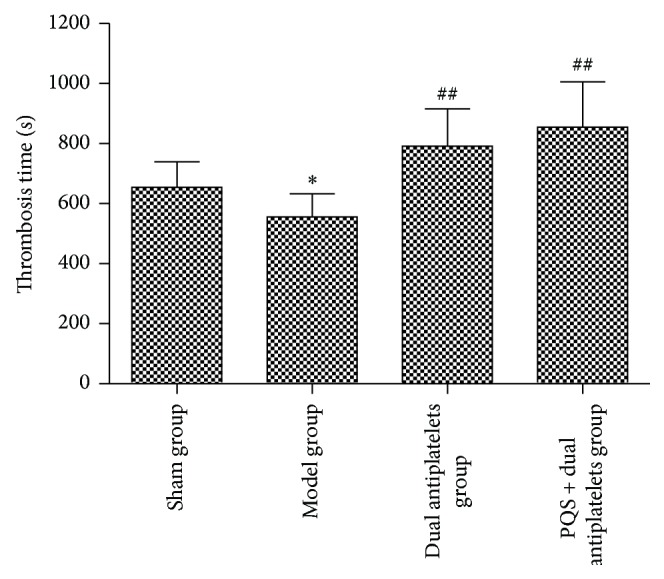
Thrombosis time in different groups. The thrombosis time was significantly longer in the dual-platelets and PQS + dual antiplatelets groups than in the model group. Data are mean ± SD (*n* = 12 rats/group). ^*^ *P* < 0.05 versus sham group, ^##^ *P* < 0.01 versus model group.

**Figure 6:**
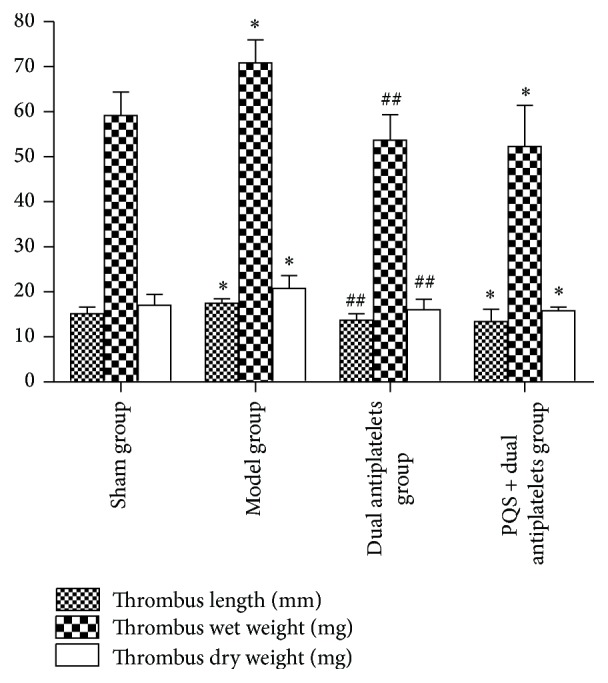
Thrombus length, thrombus wet weight, and thrombus dry weight in different groups. A decreased tendency of thrombus length, wet and dry weight* in vitro* has been observed in PQS + dual antiplatelets group versus dual antiplatelets group, but there was no significant difference between them. Data are mean ± SD (*n* = 12 rats/group). ^*^ *P* < 0.05 versus sham group, ^##^ *P* < 0.01 versus model group.

Thrombosis time* in vivo* shortened significantly (*P* = 0.031), and thrombus length, wet and dry weight* in vitro* increased significantly in model group (*P* = 0.017, *P* = 0.031, and *P* = 0.038) compared with sham group. Compared with model group, thrombosis time* in vivo* prolonged significantly and thrombus length, wet and dry weight* in vitro* decreased significantly in dual antiplatelets group (*P* = 0.013, *P* = 0.010, *P* = 0.002, and *P* = 0.003) and PQS + dual antiplatelets group (*P* = 0.003, *P* = 0.005, *P* = 0.001, and *P* = 0.002). Rats in PQS + dual antiplatelets group showed a decreased tendency in thrombosis time* in vivo* (*P* = 0.405), thrombus length (*P* = 0.817), and wet and dry weight (*P* = 0.725 and *P* = 0.861, resp.)* in vitro* as well, but no significant difference was observed compared with dual antiplatelets group (see Figures 5 and 6).

### 3.5. Concentration of Plasma TXB_2_ and 6-Keto-PGF_1*α*_

**Figure 7:**
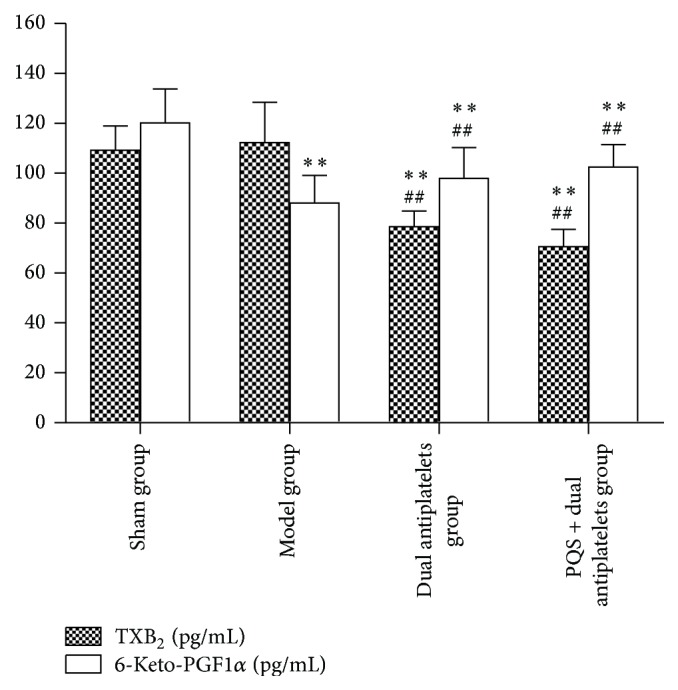
Plasma TXB_2_ and 6-keto-PGF_1*α*_ concentration in different groups. Plasma TXB_2_ concentration was significantly decreased in the dual antiplatelets group and PQS + dual antiplatelets group compared with the model group. Plasma 6-keto-PGF_1*α*_ concentration was significantly increased in the dual antiplatelets group and PQS + dual antiplatelets group compared with the model group. Data are mean ± SD (*n* = 12 rats/group); ^**^ *P* < 0.01 versus sham group, ^##^ *P* < 0.01 versus model group.

A significant decrease of 6-keto-PGF_1*α*_ concentration has been shown in model group (*P* < 0.001) compared with sham group. TXB_2_ concentration significantly decreased in dual antiplatelets and PQS + dual antiplatelets groups (*P* < 0.001, *P* < 0.001), whereas 6-keto-PGF_1*α*_ concentration significantly increased in PQS + dual antiplatelets group (*P* = 0.022) compared with model group. A decreased tendency of TXB_2_ concentration (*P* = 0.274) and an increased tendency of 6-keto-PGF_1*α*_ concentration showed (*P* = 0.539), respectively, in PQS + dual antiplatelets group compared with dual antiplatelets group (see Figure 7).

### 3.6. Coagulation Related Markers

**Figure 8:**
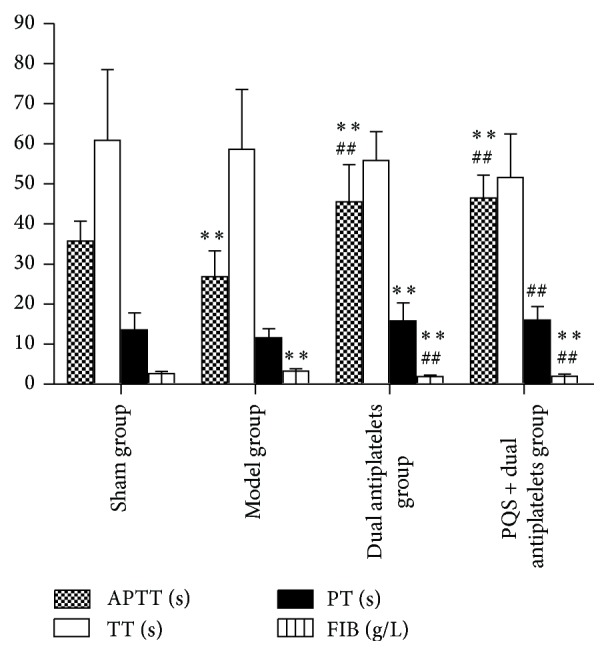
APTT, TT, PT, and FIB in different groups. APTT and PT prolonged significantly but FIB showed a significant decrease in dual antiplatelets group and PQS + dual antiplatelets group compared with model group. ^**^ *P* < 0.01 versus sham group, ^##^ *P* < 0.01 versus model group.

Activated partial thromboplastin time (APTT) shortened significantly but FIB increases significantly in model group compared with sham group (*P* = 0.002, *P* = 0.005). Compared with model group, APTT and PT prolonged significantly in dual antiplatelets group (*P* < 0.001, *P* = 0.006) and PQS + dual antiplatelets group (*P* < 0.001, *P* = 0.004), whereas higher level of FIB has been observed in two groups (*P* < 0.001, *P* < 0.001). However, there was no significant difference between antiplatelets group and PQS + dual antiplatelets group (see Figure 8).

## 4. Discussion

The Xinyue capsule, mainly composed of PQS, could significantly improve clinical symptoms of CHD patients, improving left ventricular ejection fraction (LVEF) and life quality in combination with conventional western medicine treatment [12]. Previous experiments have showed that PQS increased the levels of vascular endothelial cell growth factor (VEGF) and basic fibroblast growth factor (bFGF), thus promoting angiogenesis in ischemic myocardial area [13] and improving adenosine triphosphate (ATP) content and energy storage in ischemic cardiomyocytes as well [14]. Ischemia reperfusion injury and ventricular remodeling also have been found attenuated in rats that suffer from acute myocardial infarction [15, 16]. And this experiment showed the further beneficial effects of PQS on endothelial function, thrombosis, and platelet activity in combination with dual-platelets in rats with acute myocardial infarction.

PQS + dual antiplatelets showed a tendency in decreasing the ratio between ventricular cavity area and cardiac transverse area, a further significant decrease in plasma ET-1 concentration and a further significant increase in serum NO concentration compared with dual antiplatelets alone. PQS and dual antiplatelets showed a decreased tendency in PAgT(%), thrombosis time* in vivo*, thrombus length, wet and dry weight* in vitro*, TXB_2_ concentration, and an increased tendency in 6-keto-PGF_1*α*_ concentration compared with dual antiplatelets. In addition, there were no significant differences between PQS and dual antiplatelets and dual antiplatelets in coagulation related markers (APTT, PT, TT, and FIB).

ET had great effects on promoting vascular smooth muscle cell proliferation and vasoconstriction, whereas NO could dilate the vessel and inhibit platelet aggregation. There exists a dynamic balance between ET and NO in physiological condition. A large amount of ET release when vascular endothelium injured, which could trigger strong contraction of local vessels and facilitate platelet aggregation through binding to protein kinase isozymes and inhibiting NO release [17, 18]. In this experiment, there was a significant decrease in plasma ET-1 concentration and an increase in serum NO concentration as compared with dual antiplatelets, indicating that PQS might have beneficial effects on rats with AMI, in which process protection of vascular endothelial function and inhibition of platelet aggregation are involved. These effects result from regulation of dynamic balance between ET and NO by PQS which were similar to our previous studies [19].

Very few focuses were fixed on measuring ventricular cavity area and cardiac transverse area ratio, PAgT(%), thrombosis time* in vivo*, thrombus length, and wet and dry weight* in vitro* in previous studies. In this experiment, there was no significant difference between PQS + dual antiplatelets group and dual antiplatelets group alone. However, the platelet activity, antithrombotic activity, and plasma clotting time are significantly influenced by PQS when distilling PQS in plasma directly [20]. This might be because the rats were not be given enough PQS, or injection not for oral agent should be used.

TXA_2_ greatly accelerates platelet aggregation and vasoconstriction, while PGI_2_ could prolong platelet aggregation and promote vasodilation. Because of the short half-life period and unstable characteristics of them, the concentrations of their metabolic products, TXB_2_ and 6-keto-PGF_1*α*_, were measured indirectly. Thromboxane synthetase and prostaglandin synthetase existed in platelet and vascular endothelial cell, respectively. Once vascular endothelial cells injured, platelet aggregation easily occurs due to the local PGI_2_ decrease. In this experiment, TXB_2_ concentration showed a significant decrease and 6-keto-PGF_1*α*_ concentration showed a significant increase in PQS + dual antiplatelets group compared with model group, which indicated that PQS + dual antiplatelets might prolong platelet aggregation through regulating the production or dynamic balance of TXB_2_ and 6-keto-PGF_1*α*_, but rats in PQS + dual antiplatelets group showed no significant difference compared with dual antiplatelets group on TXB_2_ and 6-keto-PGF_1*α*_. However, previous study [21] had showed PQS 25 mg/kg, 50 mg/kg significantly decrease the 6-keto-PGF_1*α*_ concentration and significantly increase the TXB_2_ concentration, and the injection of PQS might make the differences.

In conclusion, combining with dual antiplatelets, PQS might improve vascular endothelial function and protect ventricular remodeling in rats with AMI. Although PQS + dual antiplatelets treatments have shown tendency of better antithrombotic effect compared to dual antiplatelets alone, there is no significant difference. Therefore, the antithrombotic effect of combination of PQS and dual antiplatelets treatment will be further investigated, while other forms of PQS such as injection will be used in our future study.

## 5. Conclusion

We have provided experimental evidence supporting our conclusion that, combining with dual antiplatelets, PQS might improve vascular endothelial function and protect ventricular remodeling in rats with AMI.

## Abbreviations

PQS: * Panax quinquefolius* saponin
CHD: Coronary heart disease
AMI: Acute myocardial infarction
LAD: Left anterior descending coronary artery
NO: Nitric oxide
TXB_2_: Thromboxane B_2_
APTT: Activated partial thromboplastin time
PT: Prothrombin time
TT: Thrombin time
FIB: Fibrinogen
PCI: Percutaneous coronary intervention
CHM: Chinese herbal medicine
WM: Western medicine
ET-1: Endothelin-1
ADP: Adenosine diphosphate
PAgT(%): Platelet aggregation rate
LVEF: Left ventricular ejection fraction
SD: Standard deviation.
